# The *Interleukin-6* Gene Promoter Polymorphism -174 and Atherosclerotic Events in Overweight Transplanted Patients

**DOI:** 10.1155/2011/803429

**Published:** 2011-06-01

**Authors:** Jamal Bamoulid, Cécile Courivaud, Marina Deschamps, Béatrice Gaugler, Pierre Tiberghien, Jean-Marc Chalopin, Philippe Saas, Didier Ducloux

**Affiliations:** ^1^INSERM, UMR645, 25020 Besançon, France; ^2^Université de Franche-Comté, Faculté de Médecine et de Pharmacie, 25020 Besançon, France; ^3^Institut Fédérative de Recherche, IFR133, 25000 Besançon, France; ^4^CHU Saint Jacques, Department of Nephrology, Dialysis, and Renal Transplantation, 25030 Besançon, France; ^5^EFS Bourgogne Franche-Comté, Plateforme de Biomonitoring, CIC-BT506, 25020 Besançon, France; ^6^CHU Saint Jacques, CIC Biothérapie, CIC-BT 506, 25030 Besançon, France

## Abstract

Chronic inflammation plays a pivotal role in atherosclerosis. We hypothesized that combining overweight and a greater genetic capacity to produce IL-6 predicted by *IL-6* gene promoter polymorphism at position -174 (G→C) may allow to identify individuals exhibiting higher IL-6 and C-reactive protein (CRP) concentrations with a higher risk of atherosclerotic events (AE). The occurrence of AE was analyzed with respect to body mass index, *IL-6* gene promoter polymorphism at position -174 (G→C), and other relevant risk factors, retrospectively, in 217 renal transplant recipients and, prospectively, in 132. Circulating IL-6 concentrations were closely related to BMI (*r* = 0.55, *P* = .0005). In overweight patients, serum IL-6 concentration was found to be significantly lower in C carriers than in GG patients (4.2 [1.0–5.1] versus 7.3 pg/mL [4.4–100]; *P* = .025). The incidence of AE was higher in overweight GG patients (29.5% versus 10.1%; *P* = .0003). In multivariate analysis, overweight-GG had an increased risk to develop AE (HR 2.96 [95% CI 1.09–8.04], *P* = .034 in the retrospective cohort, and HR 2.99 [95% CI 0.92–9.33], *P* = .069 in the prospective cohort). All these data are consistent with a role for both genetic and environmental determinants of inflammation (white adipose tissue mass) in the development of AE in renal transplanted patients.

## 1. Introduction

Chronic inflammation plays a pivotal role in the pathogenesis of atherosclerosis. For instance, elevated plasma concentrations of C-reactive protein (CRP), a sensitive marker of underlying inflammation, have been shown to predict an increased risk of future cardiovascular (CV) outcomes in the general population [1]. Our group recently reported that high CRP levels were also predictive of coronary heart disease in renal transplant recipients (RTRs) [2]. Circulating CRP levels are under the control of IL-6 secretion [3]. As a result, the capacity to produce CRP, and subsequently inflammation, depends at least in part on the capacity to produce IL-6. We recently demonstrated that IL-6 concentrations are in part genetically determined and varied with *IL-6* gene promoter polymorphism at position -174 (G→C) in renal transplant recipients [4]. Moreover, it has been demonstrated that white adipose tissue—that contains adipocytes—is an important source of basal IL-6 secretion [5]. 

We hypothesized that combining overweight and a greater genetic capacity to produce IL-6 predicted by *IL-6* gene promoter polymorphism at position -174 (G→C) may allow to identify a population exhibiting higher IL-6 and CRP concentrations and carrying a higher risk of atherosclerotic events (AEs).

## 2. Materials and Methods

### 2.1. Study Design and Populations

We analyzed two groups of patients to assess whether *IL-6* gene promoter polymorphism at position -174 (G→C) may be a risk factor for the later development of AE independently of other risk factors.

The two cohorts have been previously described [4]. Briefly, we first conducted a nested genetic study in a retrospective cohort of 217 patients. These patients were transplanted between January 1990 and June 2001.

Second, we studied a prospective cohort of 132 consecutive patients transplanted between July 2001 and December 2004 in our center.

Age, gender, BMI (weight [Kg]/size^2^ [m^2^]), past history of cardiovascular disease (CVD), hypertension, prior renal transplantation, CMV donor/recipient status, HLA compatibility, and panel-reactive antibodies (PRAs) were analyzed as covariates. Dialysis mode (none, hemodialysis, or peritoneal dialysis) and its duration prior to transplantation were recorded. All parameters were collected at the transplant time. Due to the design of the first study [4], patients with pretransplant diabetes had been excluded.

Approval was obtained from the Besançon ethical committee for these studies. Informed consent was provided according to the Declaration of Helsinki.

### 2.2. Cardiovascular Risk Factors

Age, gender, weight, size, blood pressure, hemodialysis duration before transplantation, smoking status, past history of AE, immunosuppressive treatment (use of calcineurin inhibitors), and different biologic parameters were assessed upon inclusion.

### 2.3. Definition of Atherosclerotic Events

One has the following:

**(i) coronary heart disease:**

Myocardial infarction, coronary revascularization including coronary artery bypass surgery or percutaneous transluminal coronary angioplasty, and typical history of angina with abdominal coronarography or myocardial scintigraphy;

**(ii) stroke/cerebrovascular disease:**

Both nonhemorrhagic and hemorrhagic strokes and carotid endarterectomy;

**(iii) abdominal aortic or lower extremity arterial disease:**

Abdominal aortic repair, lower extremity amputation, and intermittent claudication confirmed by Doppler or arteriography findings.

### 2.4. DNA Extraction and Analysis of IL-6 Gene Promoter Polymorphism at Position -174 (G→C)

Genomic DNA (gDNA) was extracted from white blood cells using standard salting out procedure [6]. Analysis of the *IL-6* gene promoter loci was then studied using a PCR-based genotyping assay [7]. Methods have been previously described in details [4].

### 2.5. Functional Validation of the IL-6 Gene Promoter Polymorphism

The functional effects of *IL-6* gene promoter polymorphism were analyzed by measuring serum levels of IL-6, 6 months posttransplant in 36 RTR (12 carrying the CC genotype, 12 the GC genotype, and 12 the GG genotype). None of the patients experienced either acute rejection or infections at time of serum collection. Interleukin-6 was measured with high-sensitivity ELISA kits (Quantikine HS IL-6 Immunoassay, #HS600B, R&D Systems, Lille, France) according to manufacturer's instructions. Briefly, the profile of IL-6 secretion was determined according to the genotype of the promoter. Three different possible genotypes at the -174 position of the IL-6 gene promoter can then be defined and associated with a high (G/G), medium (G/C), or low (CC) secretion profile [8]. Because IL-6 is recognized as the main inducer for hepatic CRP synthesis [9], CRP (high-sensitivity determination) serum levels were measured by nephelometry (Beckman Coulter, Villepinte, France) in all patients.

### 2.6. Statistical Analysis

Arithmetic mean was calculated and expressed as ± standard deviation (SD). Circulating IL-6 concentrations were not normally distributed and their values in the three different genotypes were compared using the Kruskal-Wallis test. Differences in circulating IL-6 values between GG and C carriers were evaluated by the Mann-Whitney test. For normally distributed variables, C carriers and GG patients were compared using the *χ* ^*2*^ test for dichotomic variables and the Student *t*-test for continuous variables. Hardy-Weinberg equilibrium was assessed for the genotype distribution. Relationships between numerical variables were evaluated with the Spearman/Pearson rank test. Using log-rank tests on Kaplan Meier nonparametric estimates of the survival without atherosclerotic events distribution, we selected variables with a *P*-value lower than, or equal to,  .20. The selected variables were included into a Cox proportional hazard model, and a backward stepwise selection process was performed, this time at a classical *α* = 0.05. All baseline data were assessed on the transplant day. BMI was separated into two classes (<25 or ≥25 Kg/m^2^ corresponding to overweight patients). Results are expressed as Hazard ratio (HR) and 95% confidence interval (CI), with a *P*-value testing the null hypothesis: HR = 1. Therefore when *P*-value is less than  .05, HR is significantly different from 1. Assumptions of Cox models (log-linearity and proportionality of risk in time) were met in this analysis. Analysis was performed on Statview 5 (SAS institute Inc.).

## 3. Results

### 3.1. Study Population

#### 3.1.1. Retrospective Cohort

The cohort of 217 patients was followed for a mean duration of 11.8 ± 5.8 years posttransplant. Mean follow-up was similar in patients with the different genotypes. There was no difference in the timing of transplants between the different genotypes (data not shown). Mean age was 43 ± 13 years and 139 RTR (64%) were men. Hypertension was present in 156 RTR (72%). Mean dialysis duration was 20 ± 17 months. All the patients but five were Caucasians.

#### 3.1.2. Prospective Cohort

One hundred and thirty two patients were studied with a mean follow-up of 6.1 ± 2.8 years. Five patients died during follow-up and four lost their graft. There was no difference in the timing of transplants between the different genotypes (data not shown). Mean age was 47 ± 14 years and 86 RTR (65%) were men. Hypertension was present in 99 RTR (75%). Mean dialysis duration was 19 ± 13 months.

### 3.2. Interleukin-6 Gene Promoter^−174^ Genotype Frequencies in Renal Transplant Recipients

#### 3.2.1. Retrospective Study

Ninety five patients (43.8%) were GG homozygotes (wild type), 92 GC heterozygotes (42.4%), and 30 (13.8%) CC homozygotes (mutant) (Table 1). There was no difference in pretransplant characteristics, acute graft rejection episodes, and cumulative dose of steroid during first year of transplant between patients with the different genotypes (Table 2). The proportion of GG homozygotes was similar in patients with and without overweight (46% versus 43%). The observed allele frequencies were in Hardy-Weinberg equilibrium.

#### 3.2.2. Prospective Study

The allele frequencies were similar in the prospective cohort (GG, 42.7%; GC, 45.9%; CC, 11.4%) (Table 1). Nevertheless, in this cohort, C carriers patients were older than patients with the G allele (49 ± 13 versus 47 ± 14; *P* = .037) (Table 2). Sex ratio was also different with more females in C carriers than in GG allele carriers (47% versus 33%; *P* = .042) (Table 2). The proportion of GG homozygotes was similar in patients with and without overweight (40% versus 44%). The observed allele frequencies were in Hardy-Weinberg equilibrium.

### 3.3. Relationship between the IL-6 Gene Promoter^−174^ Genotype, Circulating IL-6, and Body Mass Index in Renal Transplant Recipients

Circulating IL-6 concentrations were closely related to BMI (*r* = 0.55,   *P* = .0005). There was a trend toward a linear increase in serum IL-6 concentrations from the CC genotype to the GG genotype (median values and ranges were, resp., 2.77 [1.02 to 9.26], 3.48 [1.11 to 76.85], and 4.19 pg/mL [2.02 to 100] in CC, GC, and GG carriers; *P* = .09). When analysis was restricted to patients with overweight, serum IL-6 concentration was found to be significantly lower in C carriers than in GG patients (4.2 [1.0–5.1] versus 7.3 pg/mL [4.4–100]; *P* = .025). By contrast, in patients without overweight, IL-6 levels were quite similar in C carriers than in GG patients (2.2 [1.4–9.3] versus 2.7 pg/mL [1.1–17.8]; *P* = .27).

CRP levels were assessed in the 217 patients of the retrospective study. Posttransplant CRP concentrations were lower in C carriers (*n* = 122) than in GG patients (*n* = 95) (3.2 ± 1.6, 4.1 ± 2.2, and 4.4 ± 2.1 mg/L in CC, GC, and GG patients, resp.; *P* = .03). Similar results were observed in the prospective cohort (*n* = 77 C carriers and *n* = 55 GG patients) (3.0 ± 1.5, 4.2 ± 2.3, and 4.7 ± 2.4 mg/L in CC, GC, and GG patients, resp.; *P* = .02).

Overweight GG patients (*n* = 27 and 17, resp., in retrospective and prospective cohorts) had higher CRP levels than other categories of patients (4.9 ± 2.3 versus 3.8 ± 2.7; *P* = .015).

These results confirmed a greater degree of inflammation in overweight GG patients. As a consequence, we compared the incidence of AE between overweight GG patients and the rest of the population.

### 3.4. Relation between IL-6 Gene Promoter Polymorphism -174, Body Mass Index, and Cardiovascular Events

#### 3.4.1. Retrospective Cohort

Mean follow-up was 11.8 ± 5.8 years.

Thirty two patients (14.7%) experienced atherosclerotic complications during the study period (Coronary Heart Disease, 24; CerebroVascular Disease, 3; Peripheral Vascular Disease, 5).

**Figure 1:**
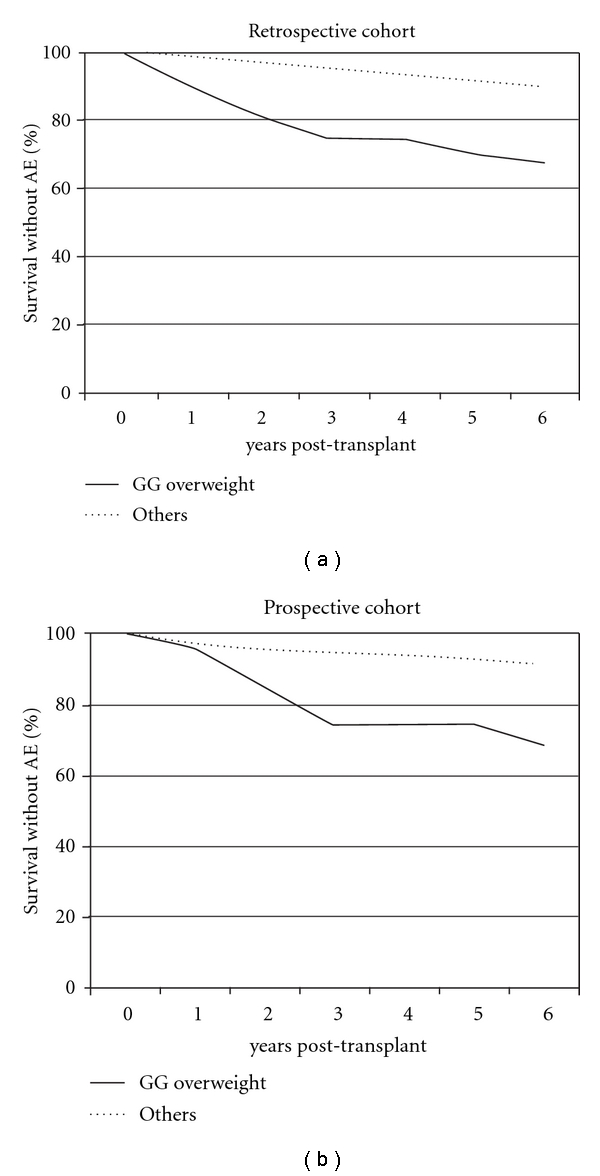
Survival without atherosclerotic events in the two cohorts.

The cumulated incidence of AE was higher in patients in overweight GG patients (33% versus 12%; *P* = .006) (Table 3 and Figure 1).

In univariate analysis, older age (*P* = .002), male gender (*P* = .031), and a past history of AE (*P* = .004) were also associated with AE.

GG patients did not carry an increased risk of AE (*P* = .251).

In multivariate analysis, older patients (HR 1.07 [95% CI 1.03–1.12] with each one year increase in age, *P* = .001) and overweight-GG patients (HR 2.96 [95% CI 1.09–8.04], *P* = .034) had an increased risk of AE (Table 4).

#### 3.4.2. Prospective Cohort

Mean follow-up was 6.1 ± 2.8 years.

Seventeen patients (11.8%) experienced atherosclerotic complications during the study period (Coronary Heart Disease, 9; CerebroVascular Disease, 3; Peripheral Vascular Disease, 5).

The cumulated incidence of AE was higher in patients in overweight GG patients (33% versus 9%; *P* = .031) (Table 3 and Figure 1).

In univariate analysis, older age (*P* = .026), male gender (*P* = .053), and a past history of AE (*P* = .004) were also associated with AE. GG patients did not carry an increased risk of AE (*P* = .251).

In multivariate analysis, older patients (HR 1.07 [95% CI 1.03–1.12] with each one year increase in age, *P* = .001) and those with a past history of AE (HR 3.88 [95% CI 1.09–11.35], *P* = .042) had an increased risk of AE. There was a trend towards an increased risk of AE in overweight GG patients (HR 2.99 [95% CI 0.92–9.33], *P* = .069) (Table 4).

#### 3.4.3. Overall Cohort

The cumulated incidence of AE was higher in patients in overweight GG patients (29.5% versus 10.1%; *P* = .0003) (Table 3). The rate of AE was similar in overweight patients than in patients with normal weight (19% versus 13.1%; *P* = .205). In patients with normal weight, the cumulative incidence of AE was similar in C carriers and GG patients (12.4% versus 14.8%; *P* = .703).

In univariate analysis, older age (*P* < .0001), male gender (*P* = .005), and a past history of AE (*P* < .0001) were also associated with AE. GG patients did not carry an increased risk of AE (*P* = .251).

In multivariate analysis, older age (HR 1.06 [95% CI 1.02–1.09] with each one year increase in age, *P* = .0005), male gender (HR 2.83 [95% CI 1.22–6.55], *P* = .016), and a past history of AE (HR 6.34 [95% CI 1.61–18.35], *P* = .008) were independent risk factors for AE. Overweight GG patients had also an increased risk of AE (HR 3.08 [95% CI 1.33–7.13], *P* = .009) (Table 4).

## 4. Discussion

Here, we reported that the GG genotype of the* IL-6* gene promoter -174, associated with a higher capacity to produce IL-6, is an independent risk factor for AE in overweight transplanted patients, but not in patients with standard weight. We also showed that IL-6 concentrations were increased only in the same subset of patients with overweight and carrying the GG genotype of the *IL-6* gene promoter at position -174. This result is probably due to the fact that white adipose tissue is a major source of basal IL-6 secretion [5]. Thus, only the combination of a greater genetically determined capacity to produce IL-6 and of an increased mass of IL-6-producing tissues seems to result in higher circulating IL-6 levels. Concordant with this result, we also found higher posttransplant CRP concentrations in overweight patients with the *IL-6* gene promoter GG genotype. Methodological standardization is mandatory in SNPs studies: a plausible hypothesis, the biological relevance of the polymorphism, and a confirmation of the results in a separate independent cohort are indispensable prerequisite in such studies [10]. All this essential conditions are met in this study. Collectively, all these data are consistent with a role for both genetic and environmental determinants of inflammation in the development of AE. 

The relationship between obesity, metabolic syndrome, and inflammation has already been described. Riikola et al. recently reported a statistical link between *IL-6* gene promoter polymorphism -174 G/C and several metabolic risk factors in men. Indeed GG genotype is associated with higher serum total cholesterol and LDL cholesterol in male [11]. This could be explained by an impaired reverse cholesterol transport *in vivo* due to inflammation as described in the study by McGillicuddy et al. [12]. Wajchenberg et al. demonstrated that low-grade inflammation is the cause of developing insulin resistance, metabolic syndrome, and endothelial dysfunction in obesity, through a chronically activated inflammatory state of the adipose tissue [13]. Moreover, we recently showed that the *IL-6* gene promoter polymorphism -174 was associated with new-onset diabetes after transplantation [4]. All these findings add to the recent notion of adipose tissue as playing a central role in lipid and glucose metabolism. Adipose tissue produces a large number of cytokines such as *IL-6* and this endocrine capacity is enhanced in obese subjects [14]. This could explain low-grade inflammation in this population which is the angular stone of metabolic syndrome and cardiovascular diseases associated with obesity. Our results are in concordance with this phenomenon. In regards of these studies, we could hypothesize that the relationship between obesity and atherosclerotic events in transplanted patients is mediated by the individual degree of inflammation at least in part genetically determined. 

Our work highlights the need to take into account plausible interactions between genetic background and environmental conditions in genetic association studies. Genetic factors are likely to affect the occurrence of numerous common diseases. Nevertheless, genes operate in complex pathways with not only gene to gene but also gene to environmental factor interactions [15]. In the specific case of IL-6, we showed that even when we showed that the IL-6 gene promoter polymorphism influences IL-6 levels in the whole RTR population, the difference is mainly explained by the difference observed in overweight patients. When considering both the GG genotype of *IL-6* gene promoter at position -174 and overweight, we defined a phenotype-genotype association with both biological and clinical relevance. Thus, we believe that an integrative approach associating both genetic and nongenetic factors is required to predict the ability to generate variable amounts of a specific gene-product and study its effects.

A selection bias is likely in the retrospective study. All the results indicate that we included low-risk patients regarding risks of AE, graft failure, and death. As a consequence, a survival bias could not be totally excluded. A differential rate of graft and patient survival or DNA availability between patients with the C or G alleles may also have influenced our results. Nevertheless, all these limitations should favor the null hypothesis and not contribute to a false positive result. Moreover, due to the design of the initial study, patients with pretransplant diabetes were excluded. Whether our results are applicable to diabetic patients remains to be determined. By the nature of the recruitment process, clinical information obtained prospectively is usually of higher quality than retrospective data and our retrospective study can only be used to generate hypotheses that have to be validated in a prospective cohort. We validated the circulating IL-6 levels towards the genotype of IL-6 gene polymorphism only with patients from the retrospective cohort (15 GG, 15 GC, 15 CC). Indeed, this validation has already been described by Fishman et al. [8]. Our aim was to demonstrate that the effect of the SNP was also effective in transplanted patients under immunosuppressive drugs. We performed the validation in the retrospective cohort. There is no reason to suspect that the association could be different in the second cohort receiving the same immunosuppressive drugs. 

Analysis of our prospective cohort confirmed the results obtained in the retrospective cohort. Yet, association of the C allele with atherosclerotic events is hardly significant in the prospective cohort (*P* = .069). The prospective cohort was smaller (*n* = 132) and the mean period of follow-up was only 6.1 ± 2.8 years. These are probably the main reasons why the association between GG genotype in overweight and atherosclerotic events (AEs) did not reach significance. It is also likely that this cohort carried a lower risk (younger age, higher percent of females). Nevertheless, it is important to note that we observed a same trend in the two cohorts allowing us to pool our data. When we pooled the two cohorts (retrospective and prospective), overweight GG genotype associated with AE reached significance (HR 3.08 [95% CI 1.33–7.13], *P* = .009).

We did not directly compared GG overweight with GG nonoverweight patients. These comparisons are made through the analysis of overweight on AE. Overweight was not associated with AE. This could be explained by the fact that a large part of the influence of overweight on AE could be due to the increased secretion of IL-6 by fat tissue in genetically predisposed patients (i.e., those carrying the GG genotype).

Finally, our results highly suggest that IL-6 production capacity influences the development of AE after kidney transplantation in overweight patients. The *IL-6* gene promoter polymorphism at position -174 may serve as a genetic marker to help physicians in determining recipient risk profile and in optimizing pre-and posttransplant treatment strategies.

## Figures and Tables

**Table 1:** ^−174^genotype frequencies in renal transplant recipients.

Genotype	Retrospective cohort		Prospective cohort	
	Number	[%]	Number	[%]
GG	95	43.8%	55	41.7%
C carriers	122	56.2%	77	58.3%
Total	217	100%	132	100%

**Table 2:** Characteristics of patients with the different *IL*-6^−174^ gene promoter genotypes.

	Retrospective cohort		Prospective cohort	
	GG	C carriers	GG	C carriers
	*n* = 95	*N* = 122	*n* = 55	*N* = 77
Age	45 ± 12	45 ± 12	47 ± 14	49 ± 13
Sex ratio (% male)	64%	64%	72%	53%
Pretransplant BMI (kg/m^2^)	23.7 ± 4.1	23.4 ± 4.6	23.9 ± 4.0	24.0 ± 4.5
Past history of cardiovascular disease	4.0%	3.9%	10.0%	12.7%
Acute rejection	22%	20%	18%	16%

BMI: body mass index.

**Table 3:** Incidence of atherosclerotic events (AEs) in the retrospective, prospective, and overall cohort according to *IL*-6^−174^ genotype and body mass index (BMI).

	All patients	Overweight patients carrying the GG genotype	Other patients	*P*
Retrospective cohort (*n* = 217)	14.7% (*n* = 32)	33% (*n* = 9)	12% (*n* = 23)	.006
Prospective cohort (*n* = 132)	11.8% (*n* = 17)	33% (*n* = 3)	9% (*n* = 12)	.031
Overall cohort (*n* = 349)	14% (*n* = 49)	29.5% (*n* = 12)	10.1% (*n* = 35)	.0003

**Table 4:** Risk factors of cardiovascular events in the retrospective, prospective, and overall cohort (multivariate analysis expressed in Hazard ratio).

	HR	IC 95%	*P*
Retrospective cohort			
Age >45 y.o	1.07	[1.03–1.12]	.001
Overweight + *IL-6* GG genotype	2.96	[1.09–8.04]	.034
Prospective cohort			
Age >45 y.o	1.07	[1.03–1.12]	.001
Past history of CVE	3.88	[1.09–11.35]	.042
Overweight + *IL-6* GG genotype	2.99	[0.92–9.33]	.069
Overall cohort			
Age >45 y.o	1.06	[1.03–1.09]	.0005
Male gender	2.83	[1.22–6.55]	.016
Overweight + *IL-6* GG genotype	3.08	[1.33–7.13]	.009
